# Could Direct Killing by Larger Dingoes Have Caused the Extinction of the Thylacine from Mainland Australia?

**DOI:** 10.1371/journal.pone.0034877

**Published:** 2012-05-02

**Authors:** Mike Letnic, Melanie Fillios, Mathew S. Crowther

**Affiliations:** 1 School of Biological, Earth and Environmental Sciences, University of New South Wales, Sydney, Australia; 2 Department of Archaeology, University of Sydney, Sydney, Australia; 3 School of Biological Sciences, University of Sydney, Sydney, Australia; University of Bern, Switzerland

## Abstract

Invasive predators can impose strong selection pressure on species that evolved in their absence and drive species to extinction. Interactions between coexisting predators may be particularly strong, as larger predators frequently kill smaller predators and suppress their abundances. Until 3500 years ago the marsupial thylacine was Australia's largest predator. It became extinct from the mainland soon after the arrival of a morphologically convergent placental predator, the dingo, but persisted in the absence of dingoes on the island of Tasmania until the 20th century. As Tasmanian thylacines were larger than dingoes, it has been argued that dingoes were unlikely to have caused the extinction of mainland thylacines because larger predators are rarely killed by smaller predators. By comparing Holocene specimens from the same regions of mainland Australia, we show that dingoes were similarly sized to male thylacines but considerably larger than female thylacines. Female thylacines would have been vulnerable to killing by dingoes. Such killing could have depressed the reproductive output of thylacine populations. Our results support the hypothesis that direct killing by larger dingoes drove thylacines to extinction on mainland Australia. However, attributing the extinction of the thylacine to just one cause is problematic because the arrival of dingoes coincided with another the potential extinction driver, the intensification of the human economy.

## Introduction

Biological invasions have been a fundamental process driving the evolution of species and shaping ecosystems through time [1]–[3]. Invasive species can impose strong selective pressures on species that have evolved in their absence, and can drive other species to extinction and re-structure ecological communities [4], [5]. This is particularly the case when the invasive species are predators that interact in novel ways with naïve prey and smaller predators [4], [5].

In communities where predators and their prey or subordinate predators have coexisted for long periods, the prey and subordinate species likely possess behaviours or morphologies that minimise the likelihood of encountering a predator or increase the chance of escape when a predator is encountered [6]. However, novel predators may have greater foraging success if prey species are naïve to their scent and foraging behaviour and thus lack effective avoidance or escape behaviour. Consequently, novel predators may have catastrophic impacts on naïve prey populations and in some cases prey on species to extinction [5].

The interactions between coexisting predators may be particularly strong, as they may compete for food, with larger predators frequently killing smaller predators and suppressing their abundances [7]–[9]. The motivation for intra-guild killing is not always predatory and larger predators may kill smaller species without eating them [10]. The relative body size of the participants is a particularly important determinant of the strength of interactions between predators. Instances of intra-guild killing tend to increase with high levels of dietary overlap, particularly when the larger species is between 2–5 times larger than the victim species with the smaller competitor almost always being the victim [7], [11]. This pattern may arise because at intermediate body size differences, the larger predator is likely to perceive the smaller species as sizeable enough to be a competitor, but small enough to defeat with minimal risk [7], [12], [13]. Carnivores that have a high dietary overlap are also likely to have more frequent encounters as they seek similar prey and hence there is greater potential for aggression or killing to occur over the contested resource. The effects of larger on smaller predators may be expected to be greater if the larger is an invasive species and the native species does not possess adaptations that help it avoid encounters with the larger predator [4].

**Figure 1:**
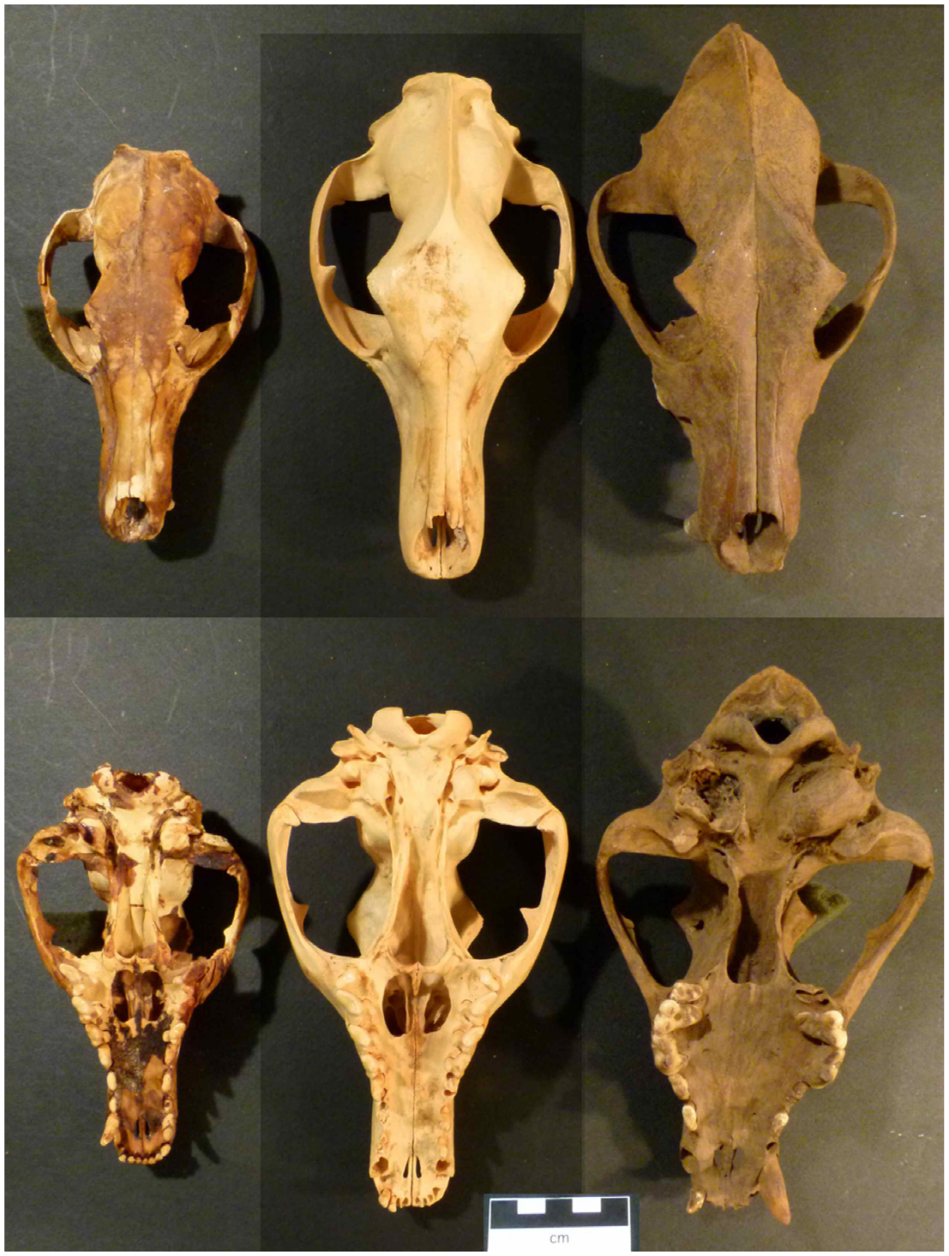
The skulls of thylacines (left WAM F6358 and centre WAM F6353) and a dingo (far right WAM 68.4.1) from sub-fossil deposits from the Nullarbor region of Western Australia. The thylacine on the far-left is thought to be a female and that in the centre a male.

Until 3500 years before present (yBP), the thylacine (*Thylacinus cynocephalus*), a marsupial, was Australia's largest terrestrial predator [14] but became extinct in mainland Australia soon after the arrival of a morphologically convergent placental predator, the dingo (*Canis lupus dingo*, Figure 1, Table S1) [15]–[17]. Because the disappearance of the thylacine and another marsupial predator, the Tasmanian devil (*Sarcophilus harrisii*), was coincident with the arrival of the dingo about 3500 yBP, some authors have suggested that dingoes caused their extinctions due to competition for food resources and confrontation with dingoes that often hunt cooperatively in packs [15], [18], [19]. However, other authors have questioned whether the dingoes could have caused the extinction of the thylacine [20], [21]. First, based on the knowledge that Tasmanian thylacines were considerably larger than dingoes (some estimates put male Tasmanian thylacines at twice the mass of dingoes), it seems unlikely that thylacines would be killed in direct confrontations with dingoes [20].

Second, based on morphological differences, thylacines and dingoes would have likely occupied very different niches. Since the thylacine was a more specialised carnivore, with a higher bite-force adjusted for allometry [15], [22]–[24], some have suggested that competition between the two species may not have been particularly intense [20]. Additional factors, such as changes in human hunting technology evident in the archaeological record, coupled with disease could also have conceivably contributed to the extinction of the thylacine from mainland Australia [20], [21], [25].

Despite speculation on dingo-thylacine interactions and the cause of the thylacine's extinction from mainland Australia, no studies have attempted to estimate the body size of Holocene thylacines from mainland Australia for comparison with dingoes. There is little information on size variation in thylacines, but there are some indications that thylacines might have become smaller in the late Pleistocene on mainland Australia, and that mainland thylacines were smaller than Tasmanian thylacines [26]–[28].

The existence of size variation between mainland and Tasmanian thylacines during the Holocene opens up the possibility that there may be more overlap in body size with dingoes, and hence potential for intra-guild killing, than has previously been considered. Here we explore the question that dingoes were responsible for the extinction of the thylacine. We do this by comparing the morphology of dingoes and mainland thylacines from the same Holocene sub-fossil deposits in mainland Australia. Our specific aim was determine if the difference in body size between mainland thylacines and dingoes was sufficient for thylacines to be susceptible to being killed by dingoes.

## Materials and Methods

We took measurements from 21 dingo and 24 thylacine specimens from Holocene deposits in temperate southwest Australia and the semi-arid Nullarbor region of Western Australia. All specimens examined are held in the palaeontology collection in the Western Australian Museum (WAM). For each specimen (Table S2) contextual information was available, including provenance, collection date, and in some cases absolute dates from radiocarbon dating.

Maturity was determined by the presence of permanent dentition, specifically full eruption of the adult dentition (i.e. third premolar in the thylacine, and eruption of molars in the dingo) [29], and synostosis of the occipito-sphenoidal suture on the skull [29]; when possible, the state of long bone epiphyseal fusion was also used, with complete fusion of the humerus, radius, ulna, femur and tibia considered to signify an adult individual (>10 months), [30].

We used measurements of the femur and humerus to estimate body mass following Anyonge [31]. On the femur, we measured the anteroposterior and mediolateral diameters at the midshafts. On the humerus, we measured the anteroposterior and mediolateral diameters at a point located at 35% of humeral length (measured from the distal end). Skull length was used as another proxy for body size and was determined by measuring the condylobasal length along the arboreal border of the occipital condyles to the prosthion. We used two-way analysis of variance (ANOVA) to compare the body mass between dingoes and thylacines at southwestern and Nullarbor locations respectively. One way ANOVA was used to compare skull length between dingoes and thylacines from the Nullarbor.

## Results

**Figure 2:**
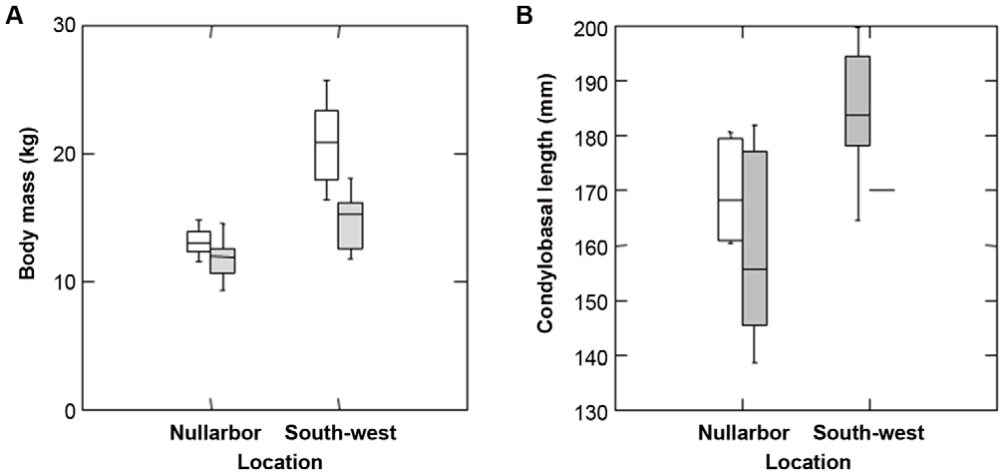
Boxplots for (A) the estimated body mass (kg) and (B) condylobasal length (mm) of dingoes and thylacines from Holocene cave deposits from the south-west and Nullarbor regions of Western Australia. Open bars denote dingoes and grey bars denote thylacines. The box indicates one quartile either side of the median, and the bars indicate two quartiles on either side of the median. The median is indicated by the bar within the box.

Based on the diameter of the limb bones, dingoes were on average heavier than thylacines (Figure 2A; F_1,21_ = 12.076, P = 0.002), although there was considerable overlap in estimated body mass, and mass in both species tended to be lower in the arid Nullarbor regions (Figure 2A; F_1,21_ = 23.87, P<0.001). A significant interaction term indicated that the difference in body weight between the two species was more marked in the mesic southwest region (Figure 2A; F_1,21_ = 4.88, P = 0.038). The smallest thylacines were 19.2% and 28.2% smaller than the smallest dingoes in the Nullarbor and southwest, respectively. The largest dingoes were estimated to be 36.8% and 54.1% heavier than the smallest thylacines in the Nullarbor and southwest, respectively.

We were only able to collect measurements from one thylacine skull in the southwest (Figure 2B). Thus comparison of condylobasal length was restricted to Nullarbor specimens, where there was no difference in the condylobasal skull length between the two species (Figure 2B; F_1,11_ = 1.32, P = 0.28). The skull of largest dingo was 23.2% longer than the smallest thylacine (Figure 2B).

## Discussion

Our measurements of indicators of body size show that there was considerable overlap in body size between thylacines and dingoes from Holocene sites in Western Australia, but on average dingoes were heavier than thylacines (Figure 2). These results support the hypothesis that dingoes would have been dominant in one on one agonistic encounters between the species owing to their larger body size [7]. Moreover, the dingo's size advantage over the thylacine may have been exacerbated by that fact that dingoes often live in packs that hunt cooperatively [32], while there is little evidence of thylacines doing the same [21], [33].

There are no complete Holocene skulls or suitable skeletal material from other locations in Australia that we are aware of that would allow us to make further estimates of thylacine body size in other geographic regions. However, measurements of tooth size from specimens collected in southeast Australia [26], [27], [34] and examination of a damaged cranial specimen from northwest Australia [35](Cape Range, ML unpublished data) indicate that the trend for mainland thylacines to be smaller than their Tasmanian conspecifics during the Holocene was not restricted to southwest Australia [26]. Our measurements showed that individuals of both species tended to be heavier in the mesic south-west region than in the arid Nullarbor region. This is consistent with previous studies that have shown body size in carnivores tends to be greater in areas with higher primary productivity and hence food availability [36]. A potential source of error affecting our body mass estimates was that the mass equations of Anyonge [31] were developed for carnivorans, but not marsupials. However, our results showed that there were consistent differences between dingoes and thylacines for both of the proxy measurements of body size (skull length and estimated body mass) that we measured.

Although there was considerable overlap in condylobasal length and estimated body mass between dingoes and thylacines, it was evident from the data that thylacines were dimorphic (Figure S1). Given that Tasmanian thylacines were sexually dimorphic, with cranial measurements of males ranging from 13 to 86% larger than that of females [37], it is likely that the dimorphism in the specimens from southwest Australia was due to size differences between the sexes. This dimorphism was likely to have had major implications for the outcomes of interactions between dingoes and thylacines. The largest thylacine specimens we measured were similarly sized to dingoes and were presumably males. Based on body size alone, large thylacines may have been capable of matching a dingo in a direct confrontation, but some of the thylacines we measured were less than half the size of dingoes from the same region. Presumably, these small, adult thylacines were females. This marked difference in size would have meant that female thylacines would have been very vulnerable to being killed in direct one-on-one encounters with dingoes.

Killing of female thylacines by dingoes could conceivably have resulted in the extinction of thylacines if it depressed the reproductive output of the thylacine population so that their rate of mortality was above the rate of the recruitment. Such a scenario does not seem implausible given that thylacines may have lacked adaptations to detect, avoid and escape invasive dingoes. Examples of larger predators suppressing the abundance of smaller predators are many [9], and the effects of larger predators tend to be exacerbated when the predator is an invasive species [4]. Another mechanism through which larger predators can affect smaller predators adversely is through aggressive interference or encounter competition; here, small predators avoid larger species, and modify their behaviour to reduce the risk of encounters with larger predators [38]–[40]. Such competition can conceivably suppress the abundance of smaller predators if it results in their access to resources being severely curtailed [41], and might be expected to occur given the size difference between dingoes and female thylacines.

Previous authors have suggested that dingoes may have driven thylacines to extinction through competition for prey [18], [19]. Metabolic rate in dasyurid marsupials is considerably lower than that of similar sized carnivores [19]. Thus, it is likely that on a per-capita basis dingoes may have needed to consume a greater mass of prey than a thylacine of a similar size. Moreover, when they first arrived in Australia, dingoes may have had a greater impact on prey populations than thylacines because they were a novel predator against which Australian prey had not evolved anti-predator defences [3], [42], [43]. Hence, competition between the two species may have been considerable. However, contemporary understanding of the processes of biological invasion and exploitative competition suggest that it is unlikely that competition with dingoes would have been the primary factor that caused the extinction of the thylacine. This is because competition has rarely been identified as the primary driver of extinction events, and is thus considered a weak extinction threat [44].

An alternative hypothesis put forward to explain the extinction of the thylacine from mainland Australia is that humans were responsible for their extinction [21], [26]. There is evidence of an increase in human population size and a shift in technology in mainland Australia around the time the thylacine went extinct [25], [45], [46]. These changes were not so evident in Tasmania where thylacines remained until the mid 20^th^ century and human technologies differed from mainland Australia [47]. Hence, it is plausible that people with the aid of new technology, and possibly using dingoes as hunting aids, were responsible for the extinction of the thylacine either through competition for prey or direct killing. Our model, that larger dingoes were likely the superior competitor in direct confrontations and therefore drove thylacines to extinction, does not exclude the idea that shifts in human technology and population size also contributed to the species' decline. Indeed, it is conceivable that both interactions with dingoes and intensification of the human economy may have both contributed to the demise of the thylacine.

### Conclusion

Dingoes were similarly sized to male thylacines but were considerably larger than female thylacines on mainland Australia during the Holocene. Small size may have made female thylacines particularly susceptible to direct killing by dingoes and such killing could have driven thylacines to extinction. Due to their lower metabolic rate and convergent morphology, thylacines would have also been susceptible to resource competition with dingoes, but competition is generally thought to be a weaker extinction threat than predation. Our results provide support for the hypothesis that direct killing by larger dingoes contributed to the extinction of the thylacine on mainland Australia. However, attributing the extinction of the thylacine to just one cause is problematic because the arrival of dingoes coincided with another potential extinction driver, the intensification of the human economy.

## Supporting Information

Figure S1 **The frequency distribution of the estimated body mass of thylacines from (A) the south-west and (B) the Nullarbor regions of Australia.**
(PDF)

Table S1 **Published radio-carbon dates of sub-fossil skeletal material (>2000 yBP) of thylacines and dingoes from mainland Australia.** Abbreviations denote South Australia (SA), Western Australia (WA) and New South Wales (NSW).
(PDF)

Table S2 **Dingo and thylacine specimens examined in the Western Australian Museum.** The date and source of the radio-carbon dated specimens is presented.
(PDF)
