# Supplementary material for: Could Direct Killing by Larger Dingoes Have Caused the Extinction of the Thylacine from Mainland Australia?
Source: PLoS One. 2012 May 2;7(5):e34877. doi: 10.1371/journal.pone.0034877 (PMC3342279; doi:10.1371/journal.pone.0034877)
Supplement: Figure S1 — The frequency distribution of the estimated body mass of thylacines from (A) the south-west and (B) the Nullarbor regions of Australia. (PDF) [file pone.0034877.s001.pdf]

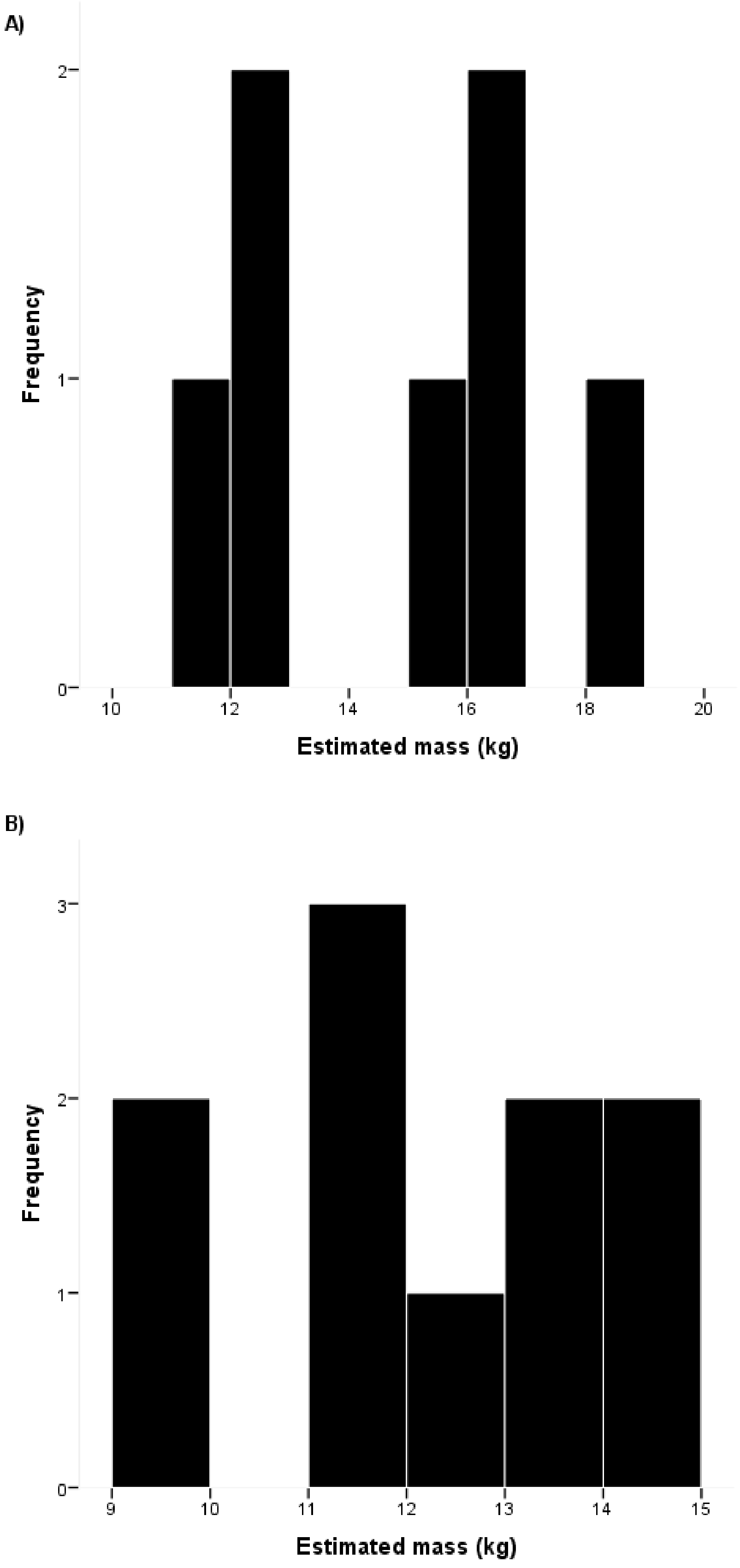

Figure. S1. The frequency distribution of the estimated body mass of thylacines from (A) the south-west and (B) the Nullarbor regions of Australia.
