# Supplementary material for: Could Direct Killing by Larger Dingoes Have Caused the Extinction of the Thylacine from Mainland Australia?
Source: PLoS One. 2012 May 2;7(5):e34877. doi: 10.1371/journal.pone.0034877 (PMC3342279; doi:10.1371/journal.pone.0034877)
Supplement: Table S1 — Published radio-carbon dates of sub-fossil skeletal material (>2000 yBP) of thylacines and dingoes from mainland Australia. Abbreviations denote South Australia (SA), Western Australia (WA) and New South Wales (NSW). (PDF) [file pone.0034877.s002.pdf]

Table S1. Published dates of sub-fossil skeletal material (> 2000 y BP) of thylacines and dingoes from mainland Australia. Abbreviations denote South Australia (SA), Western (WA) Australia and New South Wales (NSW).

Thylacine			Dingo		
Location	Date (yBP)	Source	Location	Date (yBP)	Source
Thylacine Hole,WA	4650±153	[1]	Thylacine Hole, WA	2200±96	[1]
Murra-el-elevyn Cave, WA	3280±90	[2]	Madura Cave, WA	3450±95	[3]
Fromm's Landing, SA	3881±85	[4]	Fromm's Landing, SA	3170±94	[5]
Tunnel Creek, WA	4074 ± 80, 4100 ± 67	Prior pers. comm., 2008 in [6]	Wombah, NSW	3230±100	[7]
Devon Downs Shelter, SA	3460 ± 100–2980 ± 90	[8]			
Horseshoe Cave, WA	<5630 ± 120	[9]			
Venus Bay, SA	3030±60	[10]			

References

1. Merrilees D (1970) A check on the radiocarbon dating of desiccated thylacine (marsupial "wolf") and dog tissue from Thylacine Hole, Nullarbor Region, Western Australia. Helictite 8: 39-42.
2. Partridge J (1967) A 3,300 year old Thylacine (Marsupialia: Thylacinidae) from the Nullarbor Plain, Western Australia. J R Soc West Aust 50: 57-59.
3. Milham P, Thompson P (1976) Relative antiquity of human occupation and extinct fauna at Madura Cave, southeastern Western Australia. Mankind 10: 175-180.

4. Macintosh NWG, Mahoney JA (1964) A 4,000 years old thylacine tooth (Dasyuridae) from Shelter 2. Proc R Soc Vic 77: 507-516.
5. Macintosh NWG (1964) A 3,000 years old dingo from Shelter 6. Proc R Soc Vic 77: 419-425.
6. Gale SJ (2009) Event chronostratigraphy: A high-resolution tool for dating the recent past. Quat Geochronol 4: 391-399.
7. Mulvaney DJ (1975) The Prehistory of Australia. Melbourne: Penguin Books.
8. Smith M (1982) Review of the Thylacine. In: Archer M, editor. Carnivorous Marsupials. Mosman: Royal Zoological Society of New South Wales. pp. 257-253.
9. Archer M (1974) New information about the Quaternary distribution of the thylacine (Marsupialia, Thylacinidae) in Australia. J R Soc West Aust 57: 43-49.
10. Medlin GC (1996) Report on vertebrate remains from a sinkhole in the Venus Bay Conservation Park. South Australia. Adelaide: Department of Environment and Natural Resources.
