# Supplementary material for: Could Direct Killing by Larger Dingoes Have Caused the Extinction of the Thylacine from Mainland Australia?
Source: PLoS One. 2012 May 2;7(5):e34877. doi: 10.1371/journal.pone.0034877 (PMC3342279; doi:10.1371/journal.pone.0034877)
Supplement: Table S2 — Dingo and thylacine specimens examined in the Western Australian Museum. The date and source of the radio-carbon dated specimens is presented. (PDF) [file pone.0034877.s003.pdf]

Table S2. Dingo and thylacine specimens examined in the Western Australian Museum. The date and source of radio-carbon dated specimens is presented.

Species	Registration Number	Date (yBP)	Source
Dingo	76.9.385		
Dingo	76.9.384		
Dingo	65.12.104		
Dingo	f6342/f6343*	2200±96	[1]
Dingo	63.3.25		
Dingo	63.7.163		
Dingo	63.7.163		
Dingo	64.2.40		
Dingo	b3227a		
Dingo	b3227b		
Dingo	65.12.326		
Dingo	66.2.99		
Dingo	66.2.115		
Dingo	64.2.4a		
Dingo	60.8.2		
Dingo	62.3.1		
Dingo	67.9.138		
Dingo	68.4.1		
Dingo	60.8.3		
Dingo	62.9.5		
Dingo	62.2.4b		
Thylacine	f63.55		
Thylacine	f6358		
Thylacine	f6354		
Thylacine	f6353		
Thylacine	69.12.4		
Thylacine	61.2.22		
Thylacine	70.5.5		
Thylacine	76.10.439		
Thylacine	69.7.601		
Thylacine	64.8.1	3280±90	[2]
Thylacine	76.4.40		
Thylacine	75.9.15		
Thylacine	f6356		
Thylacine	61.2.25		
Thylacine	65.5.4		
Thylacine	67.12.4		
Thylacine	64.8.1		
Thylacine	61.2.34		
Thylacine	76.10.481		
Thylacine	65.12.18		
Thylacine	65.8.5		
Thylacine	76.3.1		
Thylacine	63.3.2		
Thylacine	f6357		

*The same specimen is represented by two registration numbers.

References

1. Merrilees D (1970) A check on the radiocarbon dating of desiccated thylacine (marsupial "wolf") and dog tissue from Thylacine Hole, Nullarbor Region, Western Australia. Helictite 8: 39-42.
2. Partridge J (1967) A 3,300 year old Thylacine (Marsupialia: Thylacinidae) from the Nullarbor Plain, Western Australia. J R Soc West Aust 50: 57-59.
